# Skin Cancer Knowledge, Attitudes, and Practices among Chinese Population: A Narrative Review

**DOI:** 10.1155/2018/1965674

**Published:** 2018-06-04

**Authors:** Philip M. Stephens, Brian Martin, Ghazal Ghafari, James Luong, Vinayak K. Nahar, Linda Pham, Jiangxia Luo, Marcelle Savoy, Manoj Sharma

**Affiliations:** ^1^DeBusk College of Osteopathic Medicine, Lincoln Memorial University, Harrogate, TN, USA; ^2^Center for Animal and Human Health in Appalachia, College of Veterinary Medicine, DeBusk College of Osteopathic Medicine, and School of Mathematics and Sciences, Lincoln Memorial University, Harrogate, TN, USA; ^3^Department of English, Gannan Medical University, Ganzhou, Jiangxi, China; ^4^Carter and Moyers School of Education, Lincoln Memorial University, Harrogate, TN, USA; ^5^Lon and Elizabeth Parr Reed Health Sciences Library, DeBusk College of Osteopathic Medicine, Lincoln Memorial University, Harrogate, TN, USA; ^6^Department of Behavioral & Environmental Health, School of Public Health, Jackson State University, Jackson, MS, USA

## Abstract

Skin cancers are becoming a substantial public health problem in China. Fair skin and increased exposure to ultraviolet B (UVB) rays from the sun are among the most substantial risk factors for skin cancer development, thus making the Chinese people vulnerable to this group of diseases. The purpose of this article is to present a narrative review of the knowledge, attitudes, and practices (KAP) related to skin cancers within the Chinese population. A systematic electronic search of MEDLINE (PubMed), CINAHL, ScienceDirect, and Google Scholar databases yielded nine articles that met the inclusion criteria. The review found that although sunscreen application was a commonly used method of skin protection among the general Chinese population, educational interventions enhancing current knowledge and attitudes about the effects of UVB rays on skin from undue sun exposure were limited in many smaller communities of the country. Hence, there is an essential need to design effective, evidence-based educational programs promoting sun protection behaviors in both congregated and sparsely populated areas of China.

## 1. Introduction

Skin cancers, the most commonly diagnosed of all cancers, are typically viewed as ailments affecting primarily Caucasian populations in countries such as Australia, New Zealand, Slovenia, and Norway [1–3]. However, worldwide skin cancer rates have increased over the last three decades [4]. Populations which previously had low skin cancer rates may present the biggest challenge for public health officials due to the lack of established preventative measures. The rise in the incidence of skin cancer is becoming a significant public health problem in China. Between the years of 1990 and 1999, the rate of basal cell carcinoma (BCC) in the nearly doubled from 16.0 to 31.8 per 10,000 new cases, contributing to 60% of newly diagnosed skin cancers [5]. Squamous cell carcinoma (SCC) is the second most diagnosed type of skin cancer among the Chinese population [5]. Also reflected in the overall number of cutaneous malignancies is the increased mortality rate from melanoma between 1988 and 2007 in the cities of Shanghai and Beijing [6].

The majority of the Chinese population have fair skin along with increased exposure to ultraviolet B (UVB) rays from the sun—creating a heightened risk for the development of skin cancer diseases [7, 8]. Specifically, BCC is linked with short-term burning incidents or long-term exposure of the head and neck; SCC is often related to extended periods of short- and long-term sun exposure [9]. Thus, skin cancer rate reduction warrants use of sun protective strategies to lower UV absorption. Examples of modifiable behaviors include using sunscreen, limiting direct sun exposure, and wearing protective clothing. Therefore, addressing attitudes and perceptions related to skin cancer within the Chinese population can lead to the initiation and continuation of sun protective behaviors.

Local governments in China distribute treatment expenditures but typically fail to provide money for preventative medicine [10]. Since 2004, the central government has worked to increase cancer prevention funding [10]. As China's rapidly aging population continues to strain its healthcare system financially, preventative measures are of utmost importance due to their cost-saving benefits. The importance of these actions extends beyond the elderly of China's population.

A study conducted on staff and volunteers from seven different Olympic event locations in Beijing found that 79.3% were aware of the association between UV exposure and skin cancers. Nevertheless, only 49.3% of participants wore protective clothing, 45.3% used sunglasses, and 58.8% applied sunscreen [11]. In another study, sunscreen usage rates were as low as 23.1% among college-age Chinese residents [12]. These findings suggest that more intervention regarding sun safety should be provided to young Chinese adults. Education is especially important due to a significant risk of SCC from early age exposure in males and lifetime sun exposure in females [9].

This article presents a narrative review on the knowledge, attitudes, and practices (KAP) related to skin cancers within the Chinese population. Based on this framework, recommendations of preventative public health strategies to engage the population have been made.

## 2. Methods

In the initial search, a systematic, computer-based literature search was conducted using MEDLINE (PubMed), Cumulative Index to Nursing and Allied Health Literature (CINAHL), and ScienceDirect. The search was performed in these electronic databases using combinations of the following terms: “China”, “Chinese”, “skin cancer”, “melanoma”, “sun protection”, “sun behaviors”, “knowledge”, “attitudes”, “beliefs”, “perceptions”, “sunscreen”, “prevention”, “practices”, and “behaviors”. Results of the initial literature search conducted in August 2017 included reviews of abstracts and titles with exclusion of off-topic articles.

Analyses of further studies updated in October 2017 were performed by reviewing reference lists of the articles of interest, along with searches in Google Scholar. The search was not limited by date of publication or language. Both English and Chinese language studies that contained digital and searchable English abstracts and keywords were included.

To the best of the reviewers' knowledge, all manuscripts published in peer-reviewed journals in the selected databases were considered for inclusion in this review. Research studies that measured skin cancer or sun protection related knowledge, attitudes, beliefs, and behaviors in China were included. Exclusion criteria for the articles included the following: (1) irrelevant topics to review article aim, (2) articles that focused on treatment of skin cancers instead of preventative practices, and (3) comparable studies conducted on similar groups not indigenous to China. The literature search was conducted by four independent reviewers. Any disagreement regarding inclusion criteria was resolved via discussion until consensus was reached.

## 3. Results

**Figure 1:**
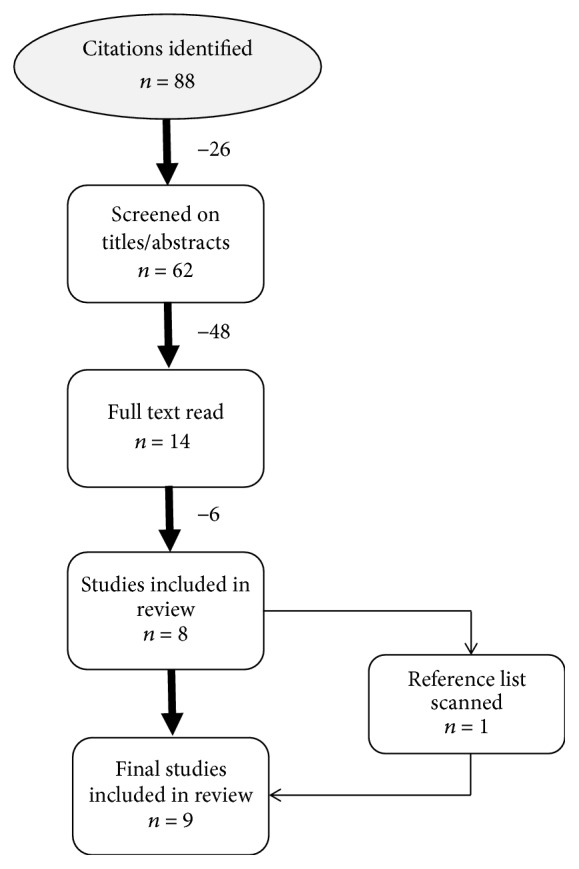
Chart of literature review.

Electronic searches identified a total of 88 citations. After removing duplicates (*n* = 26), the remaining 62 articles were screened based on titles and abstracts. After screening, the remaining 14 articles were read in their entirety to determine inclusion criteria eligibility. In summary, a total of nine articles met the eligibility criteria and were included in the review (Figure 1).

Table 1 provides details of the literature review pertaining to skin cancer knowledge, attitudes, and practices among the Chinese population. The first column displays the senior author of each corresponding article, as well as the date and location of the study. Column two includes the methodologies used for data collection and sample size (*n*) for each study, along with the gender and age of the participants. Extracted data regarding knowledge, attitudes, and beliefs of the participants are located within the third column. Only preintervention data were extracted and included in the table for studies utilizing experimental design. Prevention practices of participants in each study are described in the last column.

## 4. Discussion

The purpose of this article was to conduct a narrative review to summarize the knowledge, attitudes, and practices (KAP) related to skin cancers within the Chinese population. The Chinese are generally fair-skinned thus making them vulnerable to cutaneous cancers. The other primary modifiable preventative risk factor for skin cancers is exposure to UVB radiation, especially from the sun. The best protective measure, therefore, is limiting exposure to sunlight, particularly during the peak hours between 10 am and 2 pm. Other methods suggested to reduce skin cancer incidence include covering the skin with protective clothing—sunglasses, long sleeves, and hats—and the regular application of sunscreens with a sufficiently high sun protection factor (SPF) [8]. Sunscreen use was found to be a popular method of skin protection due to its enhanced affordability in an improving Chinese economy [11]. However, these findings were not in agreement with the results from a survey conducted among 11 communities in Shanghai, where only 21.3% utilized sunscreen, while the majority of the other participants preferred shade or hats as their primary means of skin protection [12].

While the prevalence of skin cancer is lower in China relative to some of the Western countries [11], BCC and SCC remain common cancers; therefore, skin protection remains important for public health [12]. One possible explanation for the reduced incidence of skin cancer may be that the average complexion among the Chinese populace is not as fair as that found in Western countries. One study found that the majority of the Chinese population is skin type IV on the Fitzpatrick scale, making them amenable to easy tanning and rare burning [12]. Darker-skinned populations have a lower incidence of skin cancer in general but are likely to be diagnosed with more advanced stages of the disease, if it does develop [12]. Another possible explanation may be that, in contrast to Western populations, many Chinese citizens find paler complexions more desirable and are therefore more likely to avoid unnecessary sunlight exposure and tanning [13].

Studies have found educational programs to reduce rates of sunburn and skin aging in the general population [11]. However, based on this review we found that such initiatives are rather few in China, with the effects of UVB being largely ignored [12]. Furthermore, knowledge and attitudes pertaining to UVB radiation have been found to be somewhat deficient among many Chinese communities. Among men and women, one study found that only 55.2% knew that UV radiation causes skin cancer [12]. Similar to Western populations, women scored higher on skin protective knowledge tests than men [11, 12]. Men were found to be less likely to care for their skin regularly compared to women, with many men expressing that sunscreen and umbrella usage are women's activities. Men were also less willing to participate in educational programs about skin protection [11]. Additional efforts should be made to improve the population's perspective, particularly men, on skin cancer prevention and to increase their involvement.

Sun exposure during childhood has been correlated with skin cancer development later in life [14]. The percentage of children with a history of experiencing a sunburn increases as age increases. Therefore, it is imperative for educational programs to be introduced at an early age before multiple sunburns have been manifested. Interestingly, a large percentage (93.6%) of parents reported utilizing sunscreen for skin protection on their children (including 38.2% who are less likely to utilize the same methods for themselves), citing sunburn prevention as the main reason [14]. Involving parents in their children's educational program presents a possible avenue for improving adult attitudes as well.

The appropriate application of sunscreens is another area of concern. Frequently, inadequate amounts of sunscreens were found to be used in addition to inconsistent methods of application, i.e., some areas of the body received more sunscreen, leaving other areas at risk. An application density of 2 mg/cm^2^ has been found to be most appropriate to receive the full SPF of the sunscreen [15]. Parents who did not use sunscreens on their children were fearful either that the products were unhealthy or that they would cause an allergic reaction, but among those who did use this preventative measure, nearly half did not follow the recommended reapplication rate of every 2-3 hours or used a product strength that was often less than 30 SPF [14].

Before introducing UVB awareness and skin protection programs to the Chinese population, modifications should be made to assure cultural and ethical appropriateness. One such concern is the risk of vitamin D deficiency, which Asian adults face due to their complexion [16]. In terms of disseminating knowledge and awareness to the public at large, television advertisements were selected by parents as an important medium for sun protection information (52% agreement) [14]. This finding suggests that the Chinese population is receiving a substantial portion of its medical information from nonmedical sources. Mass media campaigns, therefore, should attempt to involve medical professionals in their creation to improve the broad dissemination of effective skin protective strategies.

Knowledge alone has not been found to be a strong predictor of behavioral change [13]. The level of health literacy was a significant factor in the relationship between knowledge and behavior. For example, in the context of vitamin D absorption and sun exposure, individuals with high health literacy were more likely to discern appropriate amounts of sun exposure to maximize the amount of vitamin D absorption; unfortunately, poor attitudes counteracted the benefits of health literacy [17]. This suggests that any educational initiative should not only focus on increasing knowledge and health literacy, but also on improving societal attitudes, which insure the initiation and continuation of healthy behavioral changes related to skin protection and sun exposure. Thus, evidence-based theoretical approaches need to be instituted to foster effectual behavior change [18]. In present day context, fourth generation behavior changes that utilize multiple theories are in vogue [19]. One such utilization is through the multi-theory model (MTM) of health behavior change that breaks down behavior change into initiation and sustenance [20]. For initiation of sun protection behaviors attitudes that underscore advantages over disadvantages of such behavior change and behavioral confidence to use sun protection behaviors along with the changes in physical environment that support sun protection are important. In order to sustain sun protective behaviors, one needs to promote conversion of emotions toward goals for adhering to and continually practicing these behaviors, as well as mobilizing changes in social environment.

## 5. Limitations

There are limitations in the construct and the included studies of this narrative review. Some of the studies are susceptible to measurement biases due to self-reported data collection methods. Additionally, the data collected via questionnaires are subject to the recall bias. Although some of the studies were in Chinese with abstracts in English, the search was conducted solely using English language accessible electronic databases; therefore it is possible that some relevant articles were missed. Furthermore, we omitted searches within the grey literature. Finally, caution should be exercised when making generalizations of findings to the Chinese population since this review included only nine studies from six locations.

## 6. Conclusions

The findings of this study indicate a need for increasing awareness and knowledge among the Chinese population about skin cancer risk factors and institute effectual changes and interventions that promote sun protection behaviors. More research is needed with this population to gain a better understanding of attitudes and beliefs and how they could be adjusted into meaningful skin cancer prevention practices. Future studies must attempt to develop evidence-based theoretical interventions to help individuals to initiate and sustain sun protection behaviors in order to decrease their future skin cancer risk. Finally, research should take into consideration behavior change differences between gender, age, and socioeconomic status with regard to sun protection behaviors.

## Figures and Tables

**Table 1:** Summary of included studies.

First author, date, and location	Data collection method, sample size (*n*), gender, and age	Knowledge, attitudes, and beliefs	Skin cancer prevention practice
Cheng, 2008, and Beijing [21]	Questionnaire, *n* = 720 (patients and hospital staff) 424 females, mean age = 37 years (SD = 27.5), age range = 14–72 years	49.3% knew that sunscreen could protect people from both UVA and UVB radiation Groups within the populations of males, middle-aged, elderly, low-education, and people with skin phototype I and II had misunderstandings of sunscreen's recognition and application	Sunscreen application Use sunscreen very often: 40% Reapply sunscreen in burning sun: 43.3%

Yang, 2009, and Nanjing [15]	Direct observation, *n* = 39 (dermatologists, 28 attending, 11 residents), 21 females, mean age = 35.3 years, age range = 27–48 years *n* = 41 (photosensitive patients), 23 females, mean age = 49 years, age range = 19–72	Fluorescent agent detection according to skin site Dermatologists: Hairline of forehead = 51.3%, forehead = 92.3%, temples = 74.4%, cheek = 100%, nose = 92.3%, perioral = 82.1% (male = 61% female = 100% *p* < 0.01), ears, neck, hands, and wrists: 0% Photosensitive patients: Hairline of forehead = 36.6%, forehead = 100%, temples = 41.5% (male = 28% female = 57% *p* < 0.05), cheek = 100%, nose = 75.6%, perioral = 73.2%, ears = 2.4%, neck = 0%, hand and wrists = 4.9% Density of fluorescent agent (mg/cm^2^) Dermatologists: Hairline of forehead: male = 0.5 (SD = 0.31), female = 0.5 (SD = 0.38), forehead: male = 1.0 (SD = 0.38), female = 1.5 (SD = 0.02), temples: male = 0.5 (SD = 0.41), females = 1.0 (SD = 0.29), cheek: male = 1.0 (SD = 0.29), female = 1.0 (SD = 0.25), nose: male = 0.5 (SD = 0.35), female = 1.0 (SD = 0.21), perioral: male = 0.5 (SD = 0.32), female = 1.0 (SD = 0.17), ears: 0% Neck/V area of the chest = 0%, Hand and wrist: 0% Photosensitive patients: Hairline of forehead: male = 0.5 (SD = 0.47), female = 0.5 (SD = 0.44), forehead: male = 1.0 (SD = 0.33), female = 1.5 (SD = 0.36), temples: male = 0.5 (SD = 0.27), female = 0.2 (SD = 0.31), cheek: male = 1.0 (SD = 0.28), female = 1.0 (SD = 0.23), nose: male = 0.5 (SD = 0.26), female = 0.5 (SD = 0.35), perioral: male = 0.5 (SD = 0.36) female = 0.5 (SD = 0.25), ears: 0% Neck/V area of the neck: 0%, Hand and wrist: male = 0.1, female = 0	Sunscreen cream application Dermatologists: 28.2% put cream on the tip of finger 71.8% put cream in the palm of the hand and rubbed the hands together before applying to target skin sites Photosensitive patients: 17.1% put cream on the tip of finger 82.9% put cream in the palm of the hand and rubbed the hands together before applying to target skin sites

Cheng, 2010, and Beijing [11]	Questionnaire, *n* = 623 (volunteers), 61.8% female, mean age = 24.6 (SD = 6.7), age range = 18–60 years	Total knowledge score of types of UV that can damage the skin = 29.7%, men = 12.6%, women = 40.3% Total knowledge score that people should take precautions from the sun in the morning or at nightfall = 73.0%, men = 58.4%, women = 82.1% Total knowledge score that people should take precautions from the sun on a cloudy day = 58.8%, men = 44.5%, women = 67.5% Total knowledge score that UV-induced skin damage is accumulative = 80.3%, men = 71.4%, women = 85.7% Meaning of SPF total knowledge score = 61.2%, men = 51.3%, women = 67.3% Meaning of PA total knowledge score = 34.4%, men = 31.5%, women = 36.1% Know how to use sunscreen correctly 74.2%, men = 58.8%, women = 83.6% Awareness score of what types of skin damage the sun causes: burn (total = 81.2%, men = 70.2%, women = 88.1%), skin cancer (total = 79.3%, men = 73.1%, women = 83.1%), tan (total = 52.0%, men = 40.8%, women = 59.0%), skin aging (total = 65.8%, men = 53.8%, women = 73.3%), blemishes (total = 73.4%, men = 60.9%, women = 81.0%), do not know (total = 2.4%, men = 5.0%, women = 0.8%)	Sunscreen = 58.8%, men = 28.2%, women = 77.7% Protective clothing = 49.3%, men = 59.7%, women = 42.9% Hat = 42.2%, men = 39.5%, women = 43.9% Parasol (sun umbrella) = 45.4%, men = 14.3%, women = 64.7% Sunglasses = 45.3%, men = 42.9%, women = 46.8% No protection = 9.0%, men = 16.4%, women = 4.4% Have ever used sunscreen before = 80.3%, men = 59.2%, women = 93.2% Correct sunscreen use among those who have ever used sunscreen before = 45.0%, men = 14.9%, women = 56.8% Mean SPF value of sunscreen used = 27.7 (SD = 9.2), men = 30.7 (SD = 11.2), women = 26.7 (SD = 8.2) Mean Protection Grade (PA) value of sunscreen used = 2.3 (SD = 0.6), men = 2.6 (SD = 0.7), women = 2.3 (SD = 0.6)

Fan, 2012, and Hefei [22]	Questionnaire, *n* = 1501 (freshmen military cadets), 1488 males, mean age = 20.35 (SD = 2.13), age range = 15–29	78.8% and 81.7% of the subjects did not know that the UV consists of three parts and the meaning of the PA and SPF, respectively.	Sunscreen: 61.6% Long-sleeved clothing and pants: 48% Umbrella and hat: 61.8% Sunglasses: 63.8% 32.7% had been taken protective measures, and only 50 cases did a professional skin examination.

He, 2012, and Beijing and Ningxia [23]	Questionnaire, *n* = 217 (all females) 66.4% females from Beijing and 33.6% from Ningxia, mean age = 32 years (SD = 9.7), age range = 17–59 years	Despite the difference in cognition degrees between the two groups, both groups have high degrees of cognition on the damage of UV to skin Both groups have low basic UV knowledge 24.9% of the two groups have correct knowledge about types of UV 22.6% of the participants were aware that sun protection should be started as early as in one's infancy People get access to knowledge of sunburn and sun protection from the same sources: television 40.2%, magazines 27.6% 27.6% knew the meaning of SPF 6.3% knew the mean of PA	Walking in shadows: 63.6% Avoiding going out at noon: 60.8% Sunscreen: 62.2% Wearing long-sleeved t-shirts: 18.4% Hats: 26.7% Umbrellas: 53% Sunglasses: 30.9%

Yan, 2015, and Shanghai [12]	Questionnaires, *n* = 5964 (residents), 53.2% females, mean age = 43.2 years and age range = 20–60 years	Knowledge about UV-induced risk by gender Premature aging: male = 59.7%, female = 73.3% Immune suppression: male = 47.8%, female = 58.5% Skin cancer: male = 50.3%, female = 59.5% Sun protection attitudes by gender Need sun protection in winter: male = 27.8%, female = 43.1% Need sun protection indoors or in the vehicle: male = 21.9%, female = 32.6% Tanning attitudes by gender Appears healthy: male = 11%, female = 10.2% Looks attractive: male = 5.7%, female = 4.3% Not favorable: male = 13.7%, female = 38.1%	Sun exposure behavior by gender Avoid outdoor actives in strong sunlight: male = 71.2%, female = 82% Avoid extensive exposure in sunny midday: male = 72.2%, female = 84.2% Average daily sun exposure time (min) by gender 7AM–5PM: male = 111.8 (SD = 105.7), female = 82.2 (SD = 64.7) 10AM–2PM: male = 31.1 (SD = 45.1), female = 20.1 (SD = 28.9) 21.3% of the participants have applied sunscreen with 93.3% of respondents female

Zhou, 2015, and Nanjing [13]	Questionnaire, *n* = 253 (college students) standard care group = 126, self-regulation group = 127, 97.6% females, mean age = 21.26 years (SD = 1.34), and age range = 18–24 years	Intention to use sunscreen mean score Standard care group: 2.64 (SD = 1.41) Self-regulation group: 2.71 (SD = 1.09) Sunscreen action planning mean score Standard care group: 1.79 (SD = 0.89) Self-regulation group: 1.87 (SD = 0.86) Sunscreen coping planning mean score Standard care group: 1.83 (SD = 0.90) Self-regulation group: 1.74 (SD = 0.81)	Mean sunscreen use: Standard care group: 1.77 (SD = 1.15) Self-regulation group: 1.95 (SD = 1.21)

Zhou, 2016, and Nanjing [24]	Questionnaire, *n* = 515 (medical students), 73.2% females	Mean knowledge score of men = 2.41 (SD = 1.51) and women = 2.56 (SD = 1.38) out of maximum total score of 6, no gender difference among scores *p* = 0.31 Highest rate of correct responses 68.0%, lowest rate 9.6% Students that thought sun exposure was enough = 67.5% Students with negative sun exposure response = 32.5% Most common reasons among 44 male students for inadequate sun exposure: 43.2% avoiding dark skin, 18.2% no desire to go out, 13.6% skin cancer Most common reasons among 124 female students for inadequate sun exposure: 75.0% avoiding dark skin, 16.1% skin cancer, 12.1% accelerated aging Student knowledge of Vitamin D obtained from: media = 59.9%, health professionals = 43.3%, classmates and friends = 25%, parents = 8.8% 68% of students correctly knew that the human body can get vitamin D through sun exposure 3.0% of students had no desire to learn about vitamin D Female students had greater desire to learn compared to male students (88.3% versus 78.5%) Male students had greater indifferent attitude than female students (18.5% versus 8.7%)	Most students lacked sun exposure because they did not want to get tan Length of sun exposure: <15 mins/d = 6.8% 15–30 mins/d = 31.8% 30–45 min/d = 27.4% Not in sun for >45 min/day = 34.0% 82.7% of students used some sun protections Frequency of sun protection use Never: male = 49.3%, female = 5.6% Rarely: male = 29.4%, female = 23.0% Sometimes: male = 13.2%, female = 36.1% Often: male = 8.1%, female = 30.2% Always: male = 0%, female = 5.1% Types of sun protection Sunscreen: male = 33%, female = 75% Hats; male = >50%, female = 42% Umbrellas: male = 20%, female = 72%

Wan, 2016, and Guangzhou City [14]	Questionnaire, *n* = 3083 (parents/guardians and their children), 51.6% male children, children mean age = 7.70 years (SD = 2.78), children age range = 3–13 years, mean rent/guardian age = 36.72 years (SD = 5.95), 70.5% female	Reasons why parents supported their children in preventing sun exposure: 50.9% protecting from suntan, 75.6% protecting from sunburn, 15.3% preventing skin photoaging, 4.9% unclear, 3.1% other Reasons why parents did not support their children in preventing sun exposure: 23.2% UV is beneficial to the child's skin, 11.7% UV is no harm to the child's skin, 30.7% benefits of UV to the child's skin are greater than its harm, 52.5% the child's skin does not need sun protection Parent's opinions on using a different sunscreen for children than for parents: 69.7% the child's skin is different from the parent's skin, 27.7% the child's skin is easily allergic, 2.6% other Parent's opinions on the nonuse sunscreen for children: 44.4% sunscreen is not suitable for children, 27.9% do not know how to choose the sunscreen for children, 28.2% use alternative sun protection methods, 44.3% worried that the child's skin is allergic to sunscreen, 9.2% other Sources of sun protection information for increasing parents/guardians knowledge: 52% television advertisements 37.6% newspapers, 37% magazines, 31.1% friends, 28.2% books, 17.5% beauty parlors, 16.2% family members, 12.4% radio advertisements, 10.3% relatives, 10% dermatologist, 12.5% others	Mean time spent in the sunshine per day: <2 h: 49.8%, male = 47.7%, female = 52.1% 2–4 h: 43.9%, male = 44.6%, female = 43.1% >4 h: 6.3%, male = 7.7%, female = 4.8% Weather in which protective measures were taken: Sunny day: 93.6% Cloudy day: 8.3% Rainy day: 8.4% Cloudy to sunny day: 17.5% Occasions for sun protection: Travel: 57.8% Outdoor leisure activities: 56.4% Swimming: 36.5% Playing ball: 16.1% Physical education: 9.8% Others: 3.2% Stayed under shade: 12.8%, male = 12.3%, female = 13.4% Sunscreen: 38.2%, male = 30.2%, female = 46.6% Long-sleeved shirt: 27.6%, male = 23.8%, female = 31.7% Hat: 61.9%, male = 60.7%, female = 63.2% Umbrella: 53.2%, male = 40.8%, female = 66.4% Sunglasses: 26.8%, male = 25%, female = 28.7%
